# Atrial cardiopathy in young adults with embolic stroke of undetermined source: a myocardial deformation imaging analysis

**DOI:** 10.1007/s10554-022-02779-6

**Published:** 2022-12-21

**Authors:** Carla Marques Pires, Rita Silva, Bárbara Lage Garcia, Nuno Antunes, Catarina Vieira, Jorge Marques, Sandro Queirós, Vitor Hugo Pereira

**Affiliations:** 1 Department of Cardiology, Braga Hospital, Sete Fontes – São Victor, 4710-243 Braga, Portugal; 2 Department of Neurology, Braga Hospital, Sete Fontes – São Victor, 4710-243 Braga, Portugal; 3 Life and Health Sciences Research Institute (ICVS) School of Medicine, University of Minho, Campus de Gualtar, 4710-057 Braga, Portugal

**Keywords:** Left atrium, Embolic stroke of undetermined source, Left atrium appendage, Atrial cardiopathy, Myocardial deformation

## Abstract

**Background:**

Atrial cardiopathy (AC) has emerged as a potential pathological thrombogenic atrial substract of embolic stroke of undetermined source (ESUS), even in the absence of atrial fibrillation. Left atrium (LA) myocardial deformation analysis may be of value as a subclinical marker of AC and a predictor of ESUS.

**Aims:**

To compare LA mechanical function between ESUS cases and age and sex-matched controls.

**Methods:**

A single-center analytical study with case-control design was performed. Case group was composed by young patients admitted in the Neurology department from January 2017 to June 2021. Control group was composed by age and sex matched controls recruited from the community. All participants performed echocardiogram and a smaller sample underwent cardiac magnetic resonance.

**Results:**

We recruited 31 ESUS patients aged between 18 and 65 years and 31 age and sex matched controls. ESUS patients had a significantly higher prevalence of cardiovascular risk factors and patent foramen ovale (PFO). The prevalence of AC was not different between groups. Echocardiogram parameters, including strain analysis, were similar between groups, except for LA appendage (LAA) ostium variation which was significantly lower in ESUS patients (absolute: 6.5vs8.7mm, p<0.001; relative: 44.5%vs53.4%, p=0.002). After exclusion of patients with PFO, all the results were statistically similar. Regarding cardiac magnetic resonance analysis, there were no statistically significant differences between groups.

**Conclusion:**

This study shows that in our population atria cardiopathy and atrial function was not associated with ESUS.LAA structural and functional abnormalities may play a major role. The role of LAA in ESUS warrants further studies.

**Supplementary Information:**

The online version contains supplementary material available at 10.1007/s10554-022-02779-6.

## Introduction

Globally, stroke remains the second-leading cause of death, accounting for 11.6% of total deaths [1]. Cryptogenic strokes (CS), ischemic strokes (IS) with undefined etiology despite extensive evaluation, comprise about 50% of all IS in young adults [2]. Embolic stroke of undetermined source (ESUS) represents a recent subtype of CS defined as non-lacunar IS of probable embolic origin, in the absence of both major cardioembolic source and atherosclerosis, causing at least 50% of luminal stenosis in arteries proximal to the infarction area [3]. ESUS comprises about 80% of CS in young adults [4]. These patients have lower mortality and a higher recurrence rate (4.5% per year) than cardioembolic stroke patients [5–7].

Considering the initial belief that ESUS occurred due to covert atrial fibrillation (AF) it was hypothesized that patients could benefit from anticoagulation [3]. Nonetheless, RESPECT [8] and NAVIGATE [9] trials, which compared dabigatran and rivaroxaban vs acetylsalicylic acid, demonstrated a neutral effect on recurrence associated with higher bleeding concerns. Furthermore, several studies failed to establish a temporal and causal association between AF and ESUS development and AF was not detected in 66% of the ESUS population despite long-term continuous rhythm-monitoring [10]. A possible explanation may be the heterogeneity of this population, with numerous potential embolic mechanisms, overlapping each other. Indeed, approximately 66% of ESUS had more than one likely embolic source and the three most prevalent were left ventricle (LV) disease, atherosclerosis and atrial cardiopathy (AC) [6, 11]. Patent foramen ovale (PFO) with paradoxical embolism may also be a potential source [3].

Therefore, growing evidence suggests that AF is not a necessary condition for thromboembolic events, but a marker of left atrium (LA) disease, defined as AC [12, 13].

AC describes functional and structural anomalies of LA [14] and is associated with several biomarkers, namely: LA enlargement, supraventricular ectopy, increase of P-wave terminal force in lead V1 (PTFV1) and increase of serum N-terminal pro-brain natriuretic peptide (NT-ProBNP) [15]. A consensual and standardized diagnostic tool has not yet been proposed.

A sub-analysis of NAVIGATE TRIAL showed that ESUS patients with LA enlargement had lower recurrence events when treated with rivaroxaban, supporting the importance of AC [16]. Currently, ARCADIA [17]/ATTICUS [18] trials are studying the benefits of anticoagulation in this population.

In previously published studies, LA enlargement was commonly evaluated by LA anteroposterior diameter which is an inappropriate tool since enlargement is usually asymmetrical and not in an anteroposterior direction [19]. Furthermore, it does not provide information about the LA function.

A useful tool to assess myocardial function is the evaluation of myocardial deformation through strain analysis.

Given the putative association between AC and thromboembolic events, we sought to evaluate the role of LA strain as a subclinical marker of atrial cardiopathy and a predictor of ESUS.

This question is relevant as it may identify new markers of LA dysfunction, which can allow a better identification of patients that will benefit from anticoagulation therapy.

## Methods

### Study population

This is a single-center analytical study with a case–control design. The case group (“ESUS group”) was composed of patients admitted to the neurology department of Braga Hospital from January 2017 to June 2021 that met the following inclusion criteria: (1) age between 18 and 65 years; (2) non-lacunar IS of probable embolic origin with no cause identified after extensive diagnostic work-up. Patients included underwent standardized diagnostic procedures: cranial computed tomography (CT) or magnetic resonance (MR), CT or MR angiography to evaluate intracranial and extracranial arteries of the neck, 12-lead electrocardiogram (ECG), serial 24-h Holter monitoring and transthoracic echocardiogram. Patients with intra or extracranial atherosclerosis (at least 50% stenosis) and major risk cardioembolic sources (AF, atrial flutter, intracardiac thrombus or tumors, vegetations, prosthetic valves, moderate-severe mitral stenosis, recent myocardial infarction, LV aneurysm and severe LV dysfunction) were excluded.

ESUS patients included had initiated antithrombotic treatment immediately after stroke and the antithrombotic treatment decision was solely based on neurologist discretion.

Patients were age and sex-matched to ESUS-free volunteers recruited from the community in a 1:1 fashion.

All study subjects were recruited to an in-person meeting where the research team collected a detailed clinical history and a short physical examination together with a 12-lead ECG and transthoracic echocardiogram with LA strain analysis.

A smaller sample, 10 ESUS patients and age and sex-matched controls underwent cardiac MR in a second visit.

### Atrial cardiopathy

In this study AC was defined by the presence of at least one criterion, accordingly to previous literature and clinical data available:

1. PTFV1 > 5000 ms μV on 12-lead ECG. [17, 20, 21]
2. LA anteroposterior diameter of at least 47 mm (men) or 43 mm (women) on transthoracic echocardiogram. [22]

### Echocardiogram methods

The same investigator, who is trained in cardiac ultrasound, performed an echocardiogram in all study subjects, blinded to the case–control status, with a *General Electric Vivid E95* ultrasound device using M5Sc and 4 V probes.

Basic LA and LV measurements were obtained from the parasternal long-axis view. Measurements of mitral inflow and e′ velocity (mean of medial and lateral results) were obtained from the apical 4-chamber view using the pulsed-wave Doppler and tissue Doppler imaging, respectively. Tricuspid regurgitation velocity was assessed from a projection optimized to the regurgitation jet.

The presence of PFO was detected by morphological analysis and color-Doppler, namely in subcostal view, complemented by saline contrast analysis.

Maximum LA volume (LAV), end-diastole and end-systole LV volumes and LV ejection fraction (LVEF) were obtained using the 2-dimensional biplane Simpson method.

LAV was also determined using the 3-dimensional analysis. The LA was zoomed and the full cycle was reconstructed from a 6-cycle multibeat methodology. LAV was analysed in 4 stages of the cardiac cycle from the volume-time curve: end-systole LAV (LAVmax), mid-diastole LAV (after emptying phase), late-diastole LAV (before atrial contraction) and end-diastole LAV (LAVmin). LA reservoir volume (LAVmax-mid-diastole LAV), LA stroke volume (late diastole LAV-LAVmin), LA ejection fraction (LA stroke volume/late diastole LAV), LA cyclic volume (LAVmax-LAVmin) and LA passive emptying percentage [(LAVmax-late diastole LAV)/(LAVmax-LAVmin)] were subsequently calculated.

All volumes were indexed to the body surface area using the Mosteller formula [23].

Additionally, LA appendage (LAA) was also evaluated in the apical view and measured where the ostium was as large as possible, to evaluate de maximum and minimum ostium diameters and calculate LAA ostium absolute (LAA ostium maximum-LAA ostium minimum) and relative ((LAA ostium maximum-LAA ostium minimum)/LAA ostium maximum) variation (Fig. S1).

Finally, we analysed LA longitudinal strain (LAS) using STE with EchoPac software (*General Electric Healthcare, Milwaukee, WI, USA*) and ventricular end-diastole (closure of the mitral valve) as a time reference to define the zero-baseline for LAS curves [24]. We measured LAS in LA-focused 4 and 2-chamber apical views and divided it into 3 phases from the strain–time curve: LAS reservoir (LASr), defined as the difference between strain at mitral valve opening and mitral valve closure; LAS conduct (LAScd), the difference between strain at the onset of atrial contraction and mitral valve opening, LAS contraction (LASct), difference between strain at mitral valve closure and the onset of atrial contraction.

### Cardiac magnetic resonance methods

Cardiac MR imaging was performed on clinical 3T scanners (*Magnetom Verio, Siemens Healthcare*). Cine images were acquired using balanced steady-state free precession (bSSFP) sequences during breath-hold (field of view = 340 mm^2^; slice thickness = 8 mm 25% gap; repetition time = 57.86 ms; echo time = 1.12 ms; flip angle = 50°; acquisition matrix = 192 × 156) in short axis stack and the three-standard long-axis views: 2-, 3- and 4-chambers. The acquisition was ECG-gated, and the images were retrospectively reconstructed in 25 frames.

All the areas and diameters were measured using CMR42 (*Circle, Canada*).

LAS was analysed by a single investigator blinded to the case–control status, using feature-tracking with a previously validated custom MATLAB-based cardiac image analysis software, embedding the Medical Image Tracking Toolbox (MITT) [25] (Fig. S2).

LAS curves were computed and exported from 2- and 4-chambers views and the values of LA reservoir function (Ɛs), passive strain (Ɛe) and active strain (Ɛa) were manually obtained.

### Statistical analysis

Continuous variables were tested for normality using Kolmogorov–Smirnov’s test combined with histogram visual assessment. Measures of central tendency (mean or median) and dispersion (standard deviation or interquartile range) were chosen according to normality test result.

To examine differences between groups in normal variables the *t*-student test or Welsh test (if homogeneity of variances was not assumed) was used. In non-normal variables, a Mann–Whitney test was performed.

Categorical variables were expressed as relative and absolute frequencies and compared using the Chi-square test (*χ*^2^) or Fisher’s test, depending on expected values.

To assess the correlation between variables, a Spearman correlation (ρ) was performed, defining a small association for 0.1; a medium association for 0.3; and a strong association for values above 0.5. All analyses used IBM SPSS (*version 28; IBM corp., Armonk, NY*), a confidence interval of 95% and statistical significance was defined when p < 0.05.

### Ethical considerations

This study was approved by the Braga Hospital Ethical Committee and met the criteria established by the Declaration of Helsinki. All the participants provided written informed consent.

## Results

**Fig. 1:**
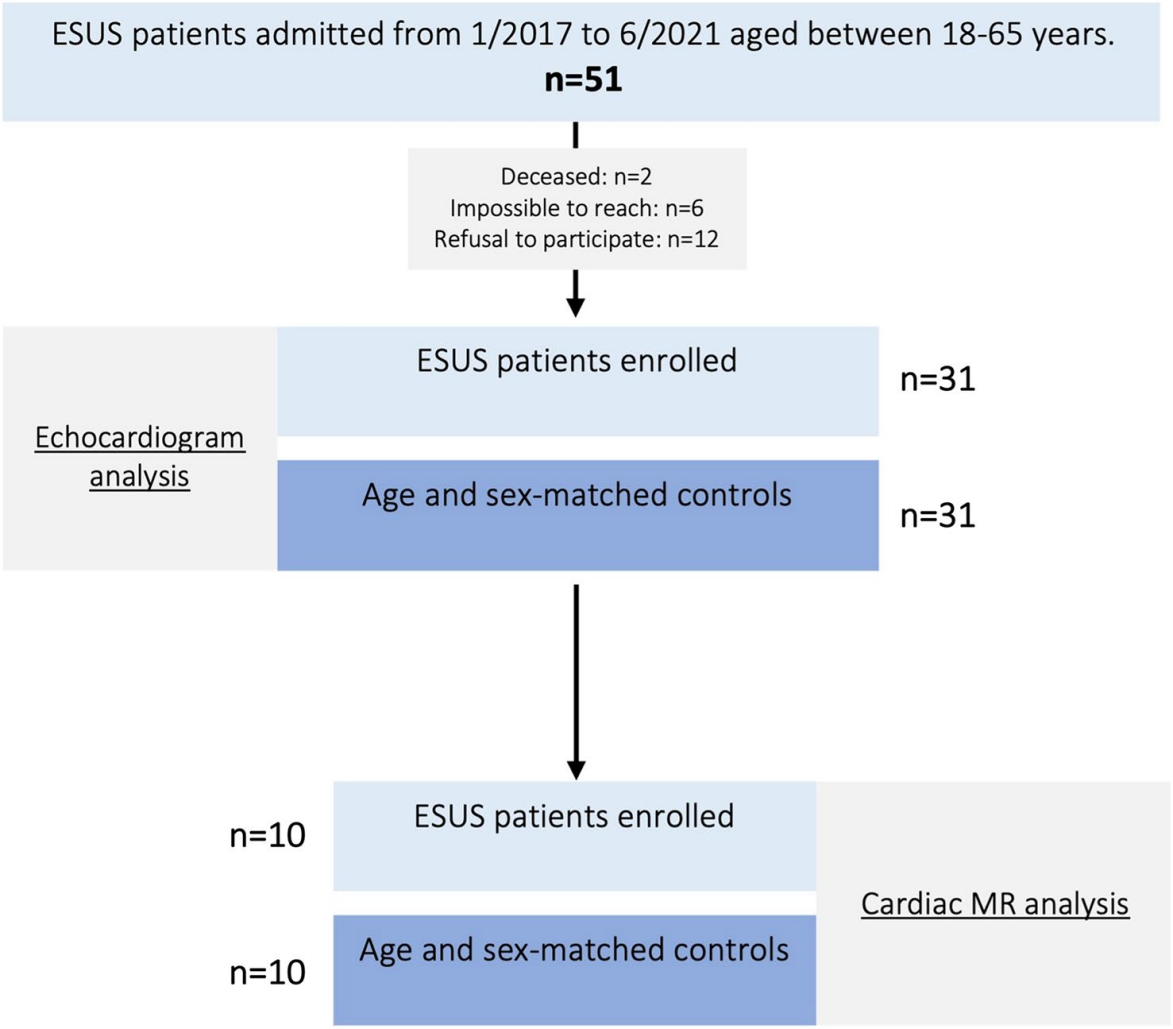
Study flow diagram

During the study period 51 patients admitted to the neurology department fulfilled the inclusion and exclusion criteria. From this pool, 31 patients accepted to be enrolled and were age and sex-matched to 31 ESUS-free controls (Fig. 1).

The mean age of the ESUS group was 50.1 ± 10.2 years (48.4% females) and of the control group was 48.0 ± 8.5 years (51.6% females). We found no significant differences regarding sex, age and anthropometric measurements. Compared with controls, ESUS patients had a higher prevalence of cardiovascular risk factors [hypertension (48.4% vs 16.1%, p < 0.001); diabetes *Mellitus* (32.3% vs 0.0%, p < 0.0001), dyslipidemia (67.7% vs 16.1%, p < 0.001) and excessive alcohol intake (25.8% vs 0.0%, p = 0.024)] and patent foramen ovale (29.0% vs 3.2%, p < 0.001).

Patients had a significantly higher usage of antiplatelets (83.9% vs 0.0%, p < 0.001), statins (83.9% vs 12.9%, p < 0.001), angiotensin-converting enzyme inhibitor/angiotensin receptor blocker (45.2% vs 9.68%, p = 0.002) and diuretics (19.4% vs 0.0%, p = 0.024).

Baseline characteristics of study subjects are presented in Table 1.

**Table 1:** Baseline clinical data of study subjects

	Patients (N = 31)	Controls (N = 31)	*P*-value
Clinical presentation			
Female gender, N (%)	15.0(48.4)	16.0(51.6)	*p* = 0.571
Age (years), mean (SD)	50.1(10.2)	48.0(8.5)	*p* = 0.548
Body mass index (kg/m^2^), median(IQR)	26.8(7.8)	25.1(4.9)	*p* = 0.328
Body surface area (m^2^), mean(SD)	1.8(0.2)	1.8(0.2)	*p* = 0.912
Abdominal circumference (cm), mean(SD)	93.3(15.1)	89.5(10.7)	*p* = 0.254
Systolic blood pressure (mmHg), median (IQR)	**133.2(16.0)**	**123.0(18.0)**	***p* = 0.005**
Diastolic blood pressure (mmHg), mean(SD)	81.2(10.7)	80.0(10.8)	*p* = 0.666
Heart rate (BPM), mean(SD)	73.1(12.5)	72.5(11.4)	*p* = 0.841
Comorbidities			
Hypertension, N(%)	**15.0(48.4)**	**5.0(16.1)**	***p* < 0.001**
Diabetes mellitus, N(%)	**10.0 (32.3)**	**0.0(0.0)**	***p* < 0.001**
Dyslipidaemia, N(%)	**21.0(67.7)**	**5.0(16.1)**	***p* < 0.001**
Smoking habits, N(%)	15.0(48.4)	10 (32.3)	*p* = 0.119
Excessive alcohol intake, N(%)	**8.0(25.8)**	**0.0(0.0)**	***p* = 0.024**
Patent foramen ovale, N(%)	**9.0(29.0)**	**1.0(3.2)**	***p* < 0.001**
Cardiovascular medication			
Antiplatelets, N(%)	**26.0(83.9)**	**0.0(0.0)**	***p* < 0.001**
Anticoagulants, N(%)	5.0(16.1)	0.0(0.0)	*p* = 0.053
Statins, N(%)	**26.0(83.9)**	**4.0(12.9)**	***p* < 0.001**
Beta-blockers, N(%)	4.0(12.9)	1.0(3.2)	*p* = 0.354
ACE inhibitors/ATR blockers, N(%)	**14.0(45.2)**	**3.0(9.7)**	***p* = 0.002**
Diuretics, N(%)	**6.0(19.4)**	**0.0(0.0)**	***p* = 0.024**
Atrial cardiopathy, N(%)	7.0(23.0)	6.0(19.0)	*p* = 0.755
Atrial cardiopathy^a^ N(%)	9.0(29.0)	7.0(23.0)	*p* = 0.772

Statistically significant results are shown in bold

*ACE* angiotensin‐converting enzyme, *ATR* angiotensin receptor blockers, *IQR* Interquartile range, *SD* standard deviation

^a^Atrial cardiopathy: using index left atrium volume instead of left atrium diameter to define moderate left atrium dilatation (> 42 ml/m^2^)

Further description of clinical characteristics of ESUS patients is presented in Table S1.

Additionally, ESUS patients were segregated according to the severity of the neurological deficit (NIHSS- National Institute of Health Stroke Scale) and there were no differences related to baseline characteristics, cardiovascular risk factors or atrial cardiopathy **(**Table S2).

### First visit

ESUS patients had a higher P-wave dispersion (25.0 ms vs 18.0 ms, p = 0.016) comparing with controls. The other electrocardiogram variables were similar between cases and controls (Table 2).

**Table 2:** Electrocardiogram performed in the visit: basic analysis

	Patients (N = 31)	Controls (N = 31)	*P*-value
Electrocardiogram			
Heart rate (BPM), mean(SD)	72.4(12.2)	70.0(11.1)	*p* = 0.531
PR interval (MS), median(IQR)	160.3(46.2)	160.2(40.0)	*p* = 0.555
P-wave mean (MS), median(IQR)	80.1(40.0)	80.2(20.1)	*p* = 0.341
P-wave max (MS), median(IQR)	100.0 (30)	94.0(28.0)	*p* = 0.495
P-wave min (MS), median(IQR)	73.0(15.5)	70.0(20.0)	*p* = 0.852
P-wave dispersion (MS), median(IQR)	**25.0(10.0)**	**19.0(18.0)**	***p* = 0.016**
P-wave axis (º), mean(SD)	41.2(25.0)	43.3(25 + 21.9)	*p* = 0.732
PTFV1 > 5000 MS ΜV, N(%)	7.0(22.6)	5.1(16.1)	*p* = 0.561

Statistically significant results are shown in bold

*IQR* interquartile range, *SD* standard deviation

LA and LV dimensions as well as LVEF did not differ significantly between groups and all study subjects had a normal diastolic function. There was a significantly lower LAA ostium variation (6.5 mm vs 8.7 mm, p < 0.001, r = 0.45) and LAA ostium relative variation (45.1% vs 53.0%, p = 0.002, d = − 0.87) in ESUS group with a strong effect size (Table 3).

**Table 3:** Echocardiogram performed in the visit: basic analysis

Echocardiogram: basic analysis	Patients (N = 31)	Controls (N = 31)	*P*-value
LV and LA basic measurements			
LVED diameter (MM), mean(SD)	45.8(4.8)	45.2(4.7)	*p* = 0.471
LVES diameter (MM), mean(SD)	29.9 (4.2)	29.1(4.1)	*p* = 0.378
IVS diameter (MM), mean(SD)	9.4(1.3)	8.7(1.8)	*p* = 0.097
LV posterior wall diameter (mm), mean(SD)	8.3(1.2)	8.2(1.4)	*p* = 0.696
LVED volume (ml/m^2^), median(IQR)	43.8(15.5)	40.8(11.0)	*p* = 0.905
LVES volume (ml/m^2^), mean(SD)	17.3(5.7)	15.9(4.3)	*p* = 0.310
LV stroke volume (ml/m^2^), median(IQR)	28.0(13.6)	27.0(8.8)	*p* = 0.387
LV ejection fraction (%), mean(SD)	62.0(7.0)	64.1(6.0)	*p* = 0.154
TAPSE (mm), mean(SD)	24.2(2.8)	24.8(2.8)	*p* = 0.408
LA diameter (mm), mean(SD)	35.5(4.8)	34.2(5.2)	*p* = 0.313
LA area (cm^2^), mean(SD)	19.0(4.3)	18.0(3.9)	*p* = 0.230
LA volume (ml/m^2^), mean(SD)	28.8(7.9)	27.5(7.6)	*p* = 0.45
LA appendage			
LAA ostium maximum (mm), mean(SD)	15.3(3.6)	16.2(2.9)	*p* = 0.279
LAA ostium minimum (mm), mean(SD)	8.6(3.2)	7.6(1.9)	*p* = 0.158
LAA ostium variation (mm), median(IQR)	**6.5(2.9)**	**8.7(2.3)**	***p* < 0.001**
LAA ostium relative variation (%), mean(SD)	**45.1(12.0)**	**53.0(7.7)**	***p* = 0.002**
Diastolic function			
E/E′, mean(SD)	7.8(3.3)	7.5(2.4)	*p* = 0.549
E′ septal (m/s), mean(SD)	0.08(0.04)	0.09(0.03)	*p* = 0.169
E′ lateral (m/s), mean(SD)	0.12(0.04)	0.11(0.03)	*p* = 0.779
Tricuspid regurgitation (m/s), mean(SD)	2.2(0.9)	2.0(0.6)	*p* = 0.200
Diastolic dysfunction, N(%)	0.0(0.0)	0.0(0.0)	–

Statistically significant results are shown in bold

*IQR* Interquartile range, *IVS* interventricular septum, *LA* left atrium, *LAA* Left atrium appendage, *LV* left ventricle, *LVED* left ventricle end-diastole, *LVES* left ventricle end-systole, *SD* standard deviation, *TAPSE* tricuspid annular plane systolic excursion

A multivariate analysis using the logistic regression model by forward method was performed and revealed that LAA ostium relative variation difference was still present (p = 0.037) after adjusting to baseline characteristics (age, gender, and cardiovascular risk factors).

**Fig. 2:**
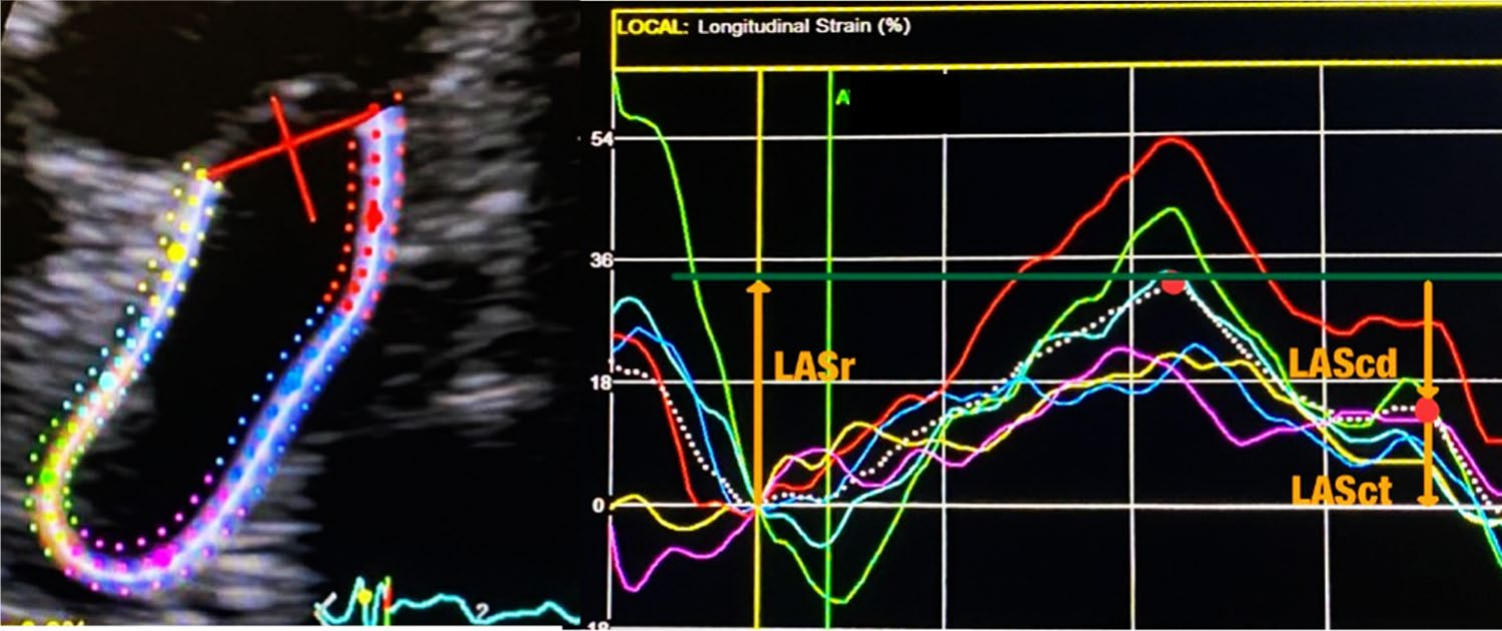
Atrial segmentation for longitudinal strain quantification using 2D echocardiography

An analysis of LA dynamics was performed through the evaluation of LA volumetry using a 4-dimensional method and myocardial deformation analysis using STE (Fig. 2). These results were shown in Table 4.

**Table 4:** Echocardiogram performed in the visit: LA dynamics analysis

Echocardiogram: LA dynamics	Patients (N = 31)	Controls (N = 31)	*P*-value
LA volumetry			
LA minimum volume (ml/m^2^), median (IQR)	12.3(6.4)	13.2(7.6)	*p* = 0.584
LA maximum volume (ml/m^2^), median (IQR)	27.4(8.4)	26.8(10.12)	*p* = 0.846
LA mid-diastolic volume (ml/m^2^), median (IQR)	17.6(7.8)	17.1(6.3)	*p* = 0.954
LA late-diastolic volume (ml/m^2^), median (IQR)	18.6(7.2)	19.0(7.0)	*p* = 0.634
LA reservoir volume (ml/m^2^), mean (SD)	8.9(2.5)	9.3(4.6)	*p* = 0.844
LA stroke volume (ml/m^2^), median (IQR)	5.0(3.8)	4.9(2.8)	*p* = 0.665
LA ejection fraction (%), median (IQR)	28.1(13.7)	26.1(14.5)	*p* = 0.745
LA cyclic volume change (ml/m^2^), median (IQR)	13.3(5.1)	12.5(7.8)	*p* = 0.445
LA conduit volume (ml/m^2^), median (IQR)	12.0(8.3)	11.4(15.1)	*p* = 0.462
LA passive emptying (%), mean (SD)	34.5(9.5)	34.1(13.5)	*p* = 0.358
LA strain/myocardial deformation analysis (4-chamber and 2-chamber)			
LASR (%), median (IQR)	42.0(16.4)	38.0(12.6)	*p* = 0.784
LASCD (%), median (IQR)	− 21.0(11.7)	− 22.0(11.7)	*p* = 0.464
LASCD (%), mean (SD)	− 18.0(5.4)	− 17.0 (4.2)	*p* = 0.270

*IQR* Interquartile range, *LA* left atrium, *LAcd-LA* longitudinal strain conduct, *LASr-LA* longitudinal strain reservoir, *LASct-LA* longitudinal strain contraction, *SD* standard deviation

There were no differences in the multiple parameters evaluated including LASr, LAScd and LASct.

Also, when analysing the ESUS patients with (n = 7.0, 22.6%) and without AC (n = 24.0, 77.4%), we found no significant differences in all LA echocardiogram findings (Table S1).

To assess the impact of PFO, an analysis without these patients was performed (Table 5). In this sub-analysis, LA and LV basic measurements and LAS were similar between groups, although a lower LAA ostium variation (6.9mm vs 8.6 mm, p = 0.003, d = − 0.89) and LAA ostium relative variation (46.0% vs 53.1%, p = 0.005, d = − 0.83) in the patient group was still observed.

**Table 5:** Study subjects without PFO: LA echocardiogram analysis

	Patients (N = 22)	Controls (N = 30)	*P*-value
LA basic measurements			
LA diameter (mm), mean(SD)	36.0(4.9)	34.0(5.2)	*p* = 0.204
LA area (cm^2^), mean(SD)	20.0(4.3)	18.0(3.9)	*p* = 0.123
LA volume (ml/m^2^), mean(SD)	29.9(8.5)	27.5(7.6)	*p* = 0.274
LA appendage			
LAA ostium maximum (mm), mean(SD)	15.1(3.5)	16.2(2.9)	*p* = 0.235
LAA ostium minimum (mm), mean(SD)	8.3(2.8)	7.6(2.0)	*p* = 0.291
LAA ostium variation (mm), mean(SD)	**6.9(2.2)**	**8.6(1.8)**	***p* = 0.003**
LAA ostium relative variation (%), mean(SD)	**46.0(11.0)**	**53.1(8.0)**	***p* = 0.005**
LA volumetry			
LA minimum volume (ml/m^2^), median (IQR)	12.7(6.3)	13.2(7.8)	*p* = 0.833
LA maximum volume (ml/m^2^), mean(SD)	27.4(5.6)	26.9(10.4)	*p* = 0.950
LA mid-diastolic volume (ml/m^2^), median (IQR)	19.0(6.1)	17.2(6.6)	*p* = 0.774
LA late-diastolic volume (ml/m^2^), mean(SD)	19.8(6.7)	19.0(7.3)	*p* = 0.786
LA reservoir volume (ml/m^2^), mean(SD)	8.8(2.9)	9.3(4.7)	*p* = 0.851
LA stroke volume (ml/m^2^), median (IQR)	4.9(4.0)	4.9(3.2)	*p* = 0.688
LA ejection fraction (%), mean(SD)	28.6(13.5)	26.7(14.4)	*p* = 0.848
LA cyclic volume change (ml/m^2^), mean(SD)	13.7(5.0)	12.7(7.6)	*p* = 0.655
LA conduit volume (ml/m^2^), mean(SD)	11.7(10.6)	11.1(15.8)	*p* = 0.256
LA passive emptying (%), mean(SD)	31.7(10.7)	34.2(13.6)	*p* = 0.782
LA strain/myocardial deformation analysis (4-chamber and 2-chamber)			
LASR (%), median (IQR)	41.0(14.4)	38.0(12.6)	*p* = 0.08
LASCD (%), median (IQR)	− 20.0(11.9)	− 23.0(11.7)	*p* = 0.210
LASCD (%), mean (SD)	− 19.0(5.5)	− 17.0(4.2)	*p* = 0.100

Statistically significant results are shown in bold

*IQR* Interquartile range, *LA* left atrium, *LAA* left atrium appendage, *LAcd-LA* longitudinal strain conduct, *LASr-LA* longitudinal strain reservoir, *LASct-LA* longitudinal strain contraction, *SD* standard deviation

### Second visit

Additionally, 10 patients and 10 age and sex-matched controls underwent cardiac MR in a second visit.

The mean age of the ESUS group was 51.1 ± 8.2 years (40.0% females) and of the control group was 49 ± 8.0 years (50.0% females). We found no significant differences regarding sex, age and anthropometric measurements.

LA basic measurements, LAA ostium variation as well as LAS did not differ significantly between groups (Table 6).

**Table 6:** Cardiac magnetic resonance analysis

	Patients (N = 10)	Controls (N = 10)	*P*-value
LA basic measurements			
LA area (cm^2^), median(IQR)	21.0(6.0)	23.0(3.0)	*p* = 0.373
LA volume (ml/m^2^), mean(SD)	30.2(9.4)	32.1(6.3)	*p* = 0.404
LA appendage			
LAA ostium maximum (mm), mean(SD)	15.1(3.6)	15.4(2.7)	*p* = 0.235
LAA ostium minimum (mm), mean(SD)	8.0(2.6)	7.7(2.6)	*p* = 0.291
LAA ostium variation (mm), mean(SD)	8.1(1.6)	7.8(1.9)	*p* = 0.710
LAA ostium relative variation (%), mean(SD)	55.0(3)	51.2(2)	*p* = 0.452
LA strain/myocardial deformation analysis (4-chamber and 2-chamber)			
Ɛs (%), mean (SD)	35.0(9.7)	38.0(9.4)	*p* = 0.426
Ɛe (%), mean (SD)	− 20.0(6.1)	− 23.0(6.4)	*p* = 0.430
Ɛa (%), median (IQR)	− 14.0(5.4)	− 16.0(3.1)	*p* = 0.283

Statistically significant results are shown in bold

*IQR* Interquartile range, *Ɛa* active strain, *Ɛe* passive strain, *Ɛs* reservoir function, *LA* left atrium, *SD* standard deviation

## Discussion

Given the putative association between AC and ESUS, the main goal of this study was to evaluate LA mechanical function using myocardial deformation analysis by STE and FT-Cardiac MR.

In line with current literature, our study showed that ESUS patients had a higher prevalence of cardiovascular risk factors [26–28] and PFO [28]. In our ESUS population 23.0% had AC, which was also consistent with published data [29], but surprisingly, compared with age and sex-matched controls the prevalence was not significantly different. In addition, LA dimensions were similar between groups.

Formerly, LA evaluation was restricted to its dimensions, however, the role of LA mechanical function in several diseases was recently highlighted and myocardial deformation analysis has been increasingly used, since it allows detection of LA dysfunction before structural changes [30].

Current evidence highlights the role of LA in ischemic strokes and recently it was used to differentiate stroke subtypes since patients with ESUS had larger LA and higher PTFV1 comparing with patients with non-cardioembolic strokes [31, 32]. In our study, a comparation between young ESUS and healthy controls was performed and, except for P-wave dispersion which was higher in cases, LA dimensions and atrium myocardial deformation analysis did not show significant differences between cases and controls. Furthermore, when analysing the ESUS patients with and without AC we found no significant differences in all LA echocardiogram findings. Considering the possibility of PFO being the source of ESUS through paradoxical embolism, we excluded these patients and repeated the analysis, but the results were similar. Considering the higher spatial resolution and better endocardial border definition of Cardiac MR [33], we also performed a LA myocardial deformation analysis using FT, which confirmed the previous findings.

Therefore, these findings suggest that in our young ESUS patients AC or LA dysfunction were not the major embolic mechanism. A possible explanation is the heterogeneity of the population which could have implications for smaller studies.

A similar case–control published in 2020, evaluated LA dynamics in 30 young CS patients (73% with ESUS) and found no differences regarding LA maximum volume, although patients had a lower epsilon peak [26]. However, in contrast with our study, LAS was evaluated using tissue Doppler imaging, which is angle-dependent and less trustworthy [34].

Moreover, a prospective cohort published in 2021, that evaluated the association of LAS using STE and stroke subtype, did not find any association with CS [35].

In addition, our study revealed a significantly lower absolute and relative LAA ostium variation in the ESUS group, which persisted after exclusion of the PFO study subjects. Furthermore, during the analysis of ESUS patients with and without AC, LAA ostium variation was similar between groups, which suggests that the LAA role may be independent of AC. In cardiac MR analysis, no differences were found in LAA ostium variation between groups. This discrepancy could be explained by a much smaller sample and flow artefacts that compromise small structure evaluation.

LAA is a remnant of the original embryonic LA and is a reservoir of blood during fluid overload [36]. Previously published data already highlighted the role of LAA in IS [14], however data in ESUS is scarce (Fig. 2).

Reduced LAA flow velocity promotes stasis, mainly if less than 20 cm/s, in AF patients [14, 37]. Additionally, 55% of CS patients had enlarged LAA [38] and non-chicken wing morphology and the number of lobes is independent risk factors of thromboembolic events [39, 40].

In summary, our study in line with current literature suggests that LAA may play a major role in ESUS pathophysiology, since lower ostium variation may hypothetically be associated with stasis and according to Virchow’s triad, thrombus formation. The role of LAA warrants further studies.

LAS did not add value for risk stratification in this group of patients.

### Limitations

This study has four main limitations. The first main limitation is that it is a single center study with a small number of participants, which restrict the statistical analysis.

The second main limitation is that most of patients lack pro-brain natriuretic peptide values data, reason why even though it is an important AC marker the authors did not included in the study.

The third main limitation is that controls did not perform 24-h Holter. Although every case had serial 24-h Holter without atrial fibrillation detection, none underwent implantable loop recorder insertion which would improve the diagnostic yield compared with 24-h Holter recording.

The last main limitation is that LA was not evaluated by computed tomography, reason why LAA morphology was not accessed.

## Supplementary Information

Below is the link to the electronic supplementary material.

Supplementary file1 (PDF 5092 kb)
