# Supplementary material for: Atrial cardiopathy in young adults with embolic stroke of undetermined source: a myocardial deformation imaging analysis
Source: Int J Cardiovasc Imaging. 2022 Dec 21;39(4):737–46. doi: 10.1007/s10554-022-02779-6 (PMC10104947; doi:10.1007/s10554-022-02779-6)
Supplement: Supplementary file 1 — Supplementary file1 (PDF 5092 kb) [file 10554_2022_2779_MOESM1_ESM.pdf]

SUPLEMENTAL MATERIAL

SUPLEMENTAL FIGURE LEGEND

Figure S1. Left atrial appendage ostium measurement (1-ostium maximum;2-ostium minimum).

Figure S2. Left atrial longitudinal strain analysis using speckle tracking echocardiography from a 4-chamber view. LAScd- conduct phase; LASct- left atrial longitudinal strain contraction phase; LASr- left atrial longitudinal strain reservoir phase.

Figure S1.

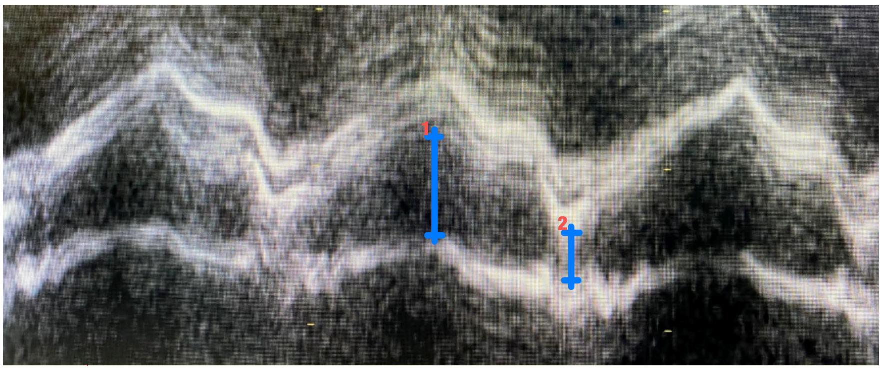

Figure S1. Left atrial appendage ostium measurement (1-ostium maximum;2-ostium minimum).

Figure S2.

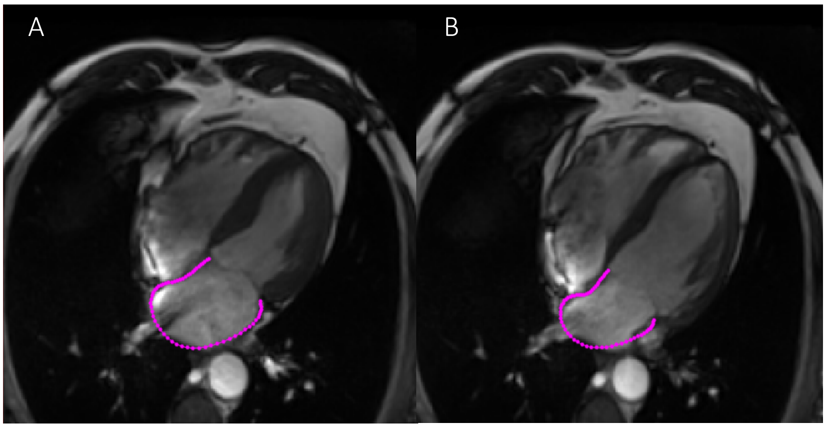

Figure S2. Left atrial longitudinal strain analysis using feature-tracking cardiac magnetic resonance from 4-chamber view.

SUPLEMENTAL TABLE LEGEND

Table S1. ESUS neurological characterization.

Table S2. ESUS characterization considering neurological deficit

Table S3. ESUS patients with and without AC: LA echocardiogram analysis.

TABLE S1.

Table S1. ESUS characterization.

	ESUS (n=31)
Female gender, n (%)	15.0(48.4)
Age (years), mean (SD)	50.1(10.2)
Body mass index (kg/m^2), median(IQR)	26.8(7.8)
NIHSS at admission, mean (SD)	5.0 (3.6)
NIHSS>5, n(%)	12.0(38.7)
OCSP classification	
PACI, n(%)	18.0(58.1)
TACI, n(%)	5.0(16.1)
POCI, n(%)	8.0(25.8)
Atrial cardiopathy*, n(%)	5.0(26.3)

IQR-Interquartile Range; NIHSS- National Institute of Health Stroke Scale, PACI-Partial Anterior Circulation Infarct; POCI - Posterior Circulation Infarct; OCSP- Oxfordshire Community Stroke Project classification; SD-Standard Deviation; TACI-Total anterior circulation Infarction.

TABLE S2.

Table S2. ESUS characterization considering neurological deficit.

	ESUS NIHSS<5 (n=19)	ESUS NIHSS≥5 (n=12)	*p-value*
**Baseline characteristic**			
Female gender, n (%)	11.0(57.9)	5.0(41.7)	*p=0.379*
Age (years), mean (SD)	50.3 (10.1)	47.8(12.6)	*p=0.536*
**Cardiovascular risk factors**			
Hypertension, n(%)	12.0(63.2)	7.0(58.0)	*p=0.238*
Diabetes mellitus, n(%)	7.0(36.8)	2.0(16.7)	*p=0.228*
Dyslipidaemia, n(%)	13.0(68.4)	8.0(66.7)	*p=0.919*
Excessive alcohol intake, n(%)	4.0(21.1)	2.0(16.7)	*P=0.574*

**Atrial cardiopathy markers**			
Atrial cardiopathy*, n(%)	5.0(26.3)	2.0(16.7)	*p=0.676*
P-wave mean (ms), mean (SD)	96.6(16.1)	106.3 (14.9)	*p=0.112*
P-wave max (ms), mean (SD)	93.1(17.1)	104.4(21.2)	*p=0.122*
P-wave min (ms), median(IQR)	70.0(16)	80.0(17.5)	*p=0.050*
P-wave dispersion (ms), mean (SD)	24.9(9.6)	27.9(8.1)	*p=0.389*
P-wave axis (º), mean(SD)	36.1(24.0)	48.6(25.6)	*p=0.190*
Abnormal P-wave axis, n(%)	3.0(15.8)	2.0(25)	*p=0.653*
PTFV1>5000ms.µV, n(%)	5(26.3)	2 (16.7)	*p=0.676*
LA diameter (mm), mean(SD)	36.6(5.2)	33.7(4.0)	*p=0.117*
LA volume (ml/m^2), mean(SD)	30.7(9.3)	28.3(5.1)	*p=0.115*

**According to definition in methods section*
IQR-Interquartile Range; NIHSS- National Institute of Health Stroke Scale; SD-Standard Deviation

TABLE S3.

Table S3. ESUS patients with and without AC: LA echocardiogram analysis.

	Patients without AC (n=24)	Patients with AC (n=7)	*p-value*
LA basic measurements			
LA diameter (mm), mean(SD)	36.1(4.5)	34.2(5.8)	*p=0.286*
LA Area (cm^2), mean(SD)	18.2(4.3)	20.3(3.9)	*p=0.273*
LA volume (ml/m^2), mean(SD)	28.8(8.3)	28.4(6.9)	*p=0.860*
LA appendage			
LAA ostium maximum (mm), mean(SD)	15.2(3.5)	15.5(4.3)	*p=0.837*
LAA ostium minimum (mm), mean(SD)	8.3(3.1)	9.4(3.7)	*p=0.468*
LAA ostium variation (mm), median(IQR)	6.9(2.4)	6.2(1.7)	*p=0.476*
LAA ostium relative variation (%), mean(SD)	42.0(10)	45.0(9)	*p=0.799*
LA volumetry			
LA minimum volume (ml/m^2), median (IQR)	12.3(11.24)	12.3(6.5)	*p*=0.207
LA maximum volume (ml/m^2), median (IQR)	27.4 (6.5)	27.3(9.6)	*p*=0.441

LA mid-diastolic volume (ml/m^2), mean(SD)	19.0(8.6)	16.3(8.7)	*p=0* .192
LA late-diastolic volume (ml/m^2), mean(SD)	20.2(9.1)	17.3(8.8)	*p*= 0.122
LA reservoir volume (ml/m^2), mean(SD)	8.3(2.6)	8.9(2.4)	*p*=0.977
LA stroke volume (ml/m^2), mean(SD)	7.3(3.4)	4.6(3.8)	*P* = 0.199
LA ejection fraction (%), mean(SD)	31.8(13.7)	27.6(14.0)	*p* =0.726
LA cyclic volume change (ml/m^2), mean(SD)	15.0(5.9)	12.7(5.0)	*p* = 0.670
LA conduit volume (ml/m^2), median (IQR)	7.0(7.4)	12.4(7.3)	*p=0.144*
LA passive emptying (%), mean(SD)	31.7(6.4)	35.9(9.1)	*p=0.382*
LA strain/myocardial deformation analysis (4-chamber and 2-chamber)			
LASr (%), median (IQR)	42.0(9.9)	37.0(10.9)	*p=0.332*
LAScd (%), median (IQR)	-23.0(7.9)	-20.0(7.0)	*p=0.290*
LAScd (%), mean (SD)	-18.0(5.7)	-18.0(4.5)	*p=0.772*

IQR-Interquartile Range; LA–Left atrium; LAA- left atrium appendage; LAcd-LA longitudinal strain conduct, LASr-LA longitudinal strain reservoir, LASct- LA longitudinal strain contraction; SD -Standard Deviation
